# Optimal Endpoint of Therapy in IBD: An Update on Factors Determining a Successful Drug Withdrawal

**DOI:** 10.1155/2015/832395

**Published:** 2015-06-14

**Authors:** Anita Annaházi, Tamás Molnár

**Affiliations:** First Department of Medicine, University of Szeged, Szeged 6720, Hungary

## Abstract

Ulcerative colitis (UC) and Crohn's disease (CD) are chronic inflammatory disorders, which require long term treatment to achieve remission and to prevent relapses and cancer. While current therapies are effective in most cases, they can have rare but serious side effects and are often associated with high costs. On the other hand, early discontinuation of an effective treatment may lead to a quick relapse and to complications at the restart of therapy. Therefore it is essential to determine the optimal duration of maintenance therapy, but clear guidelines are missing. The most important questions when deciding whether to continue or withdraw therapy in quiescent UC and CD patients are the efficacy of the continuous treatment to maintain remission in the long term, the frequency and severity of side effects, and the chance of relapse after discontinuation of therapy. This review summarizes the current knowledge on these topics with respect to 5-aminosalicylates, thiopurines, methotrexate, and biological therapies and collects information regarding when and in which specific patient groups, in the absence of risk factors, can withdrawal of therapy be considered without a high risk of relapse. Additionally, the particular aspect of colorectal cancer prevention by current therapies will also be discussed.

## 1. Introduction

Inflammatory bowel diseases (IBD), such as ulcerative colitis (UC) and Crohn's disease (CD), are chronic, immune-mediated disorders of the gastrointestinal tract. In Western countries the annual incidence rates can be as high as 6.3/100.000 for CD and 9.8/100.000 for UC, and the incidence in developing countries is also increasing [1]. The disease mostly affects individuals in their active, young and middle ages, with significant economical consequences. Currently, no curative treatment exists, but available therapies are able to suppress the abnormal immune activation, achieve remission, and prevent relapses in many patients, enabling them to lead a normal lifestyle. The efficacy of current treatments has been proven by several studies and the daily clinical practice. However, none of these medications are free from possible side effects, and many of them are associated with high costs. Therefore a new area of debate has emerged parallel to the widespread use of these medications, namely, the question of the optimal duration of treatment in IBD. According to some views, maintenance therapy should be lifelong, as in many other chronic diseases. Nevertheless, the clinical benefit of maintenance therapy in symptom-free periods is not well established, and the fear of possible side effects may incite both patients and physicians to stop therapy once remission is achieved. The most important questions in this debate are the following: is the medication able to maintain remission when taken on a regular basis or does the effect wane after a certain period of time; how often and how serious are side effects; what happens if the medication is withdrawn; and which specific risk factors are associated with relapse? This review aims to answer these questions by focusing on available information and current opinions regarding the optimal duration of therapy in UC and CD and, with the help of risk factors, aims to collect data to determine a subgroup of patients who have a very low relapse rate and where the discontinuation of therapy may be possible.

## 2. 5-Aminosalicylic Compounds

### 2.1. Efficacy of Maintenance Therapy in the Long Term

5-Aminosalicylates (5-ASA) represent the first line therapy in mild and moderate UC patients. Modern 5-ASA preparates are all related to sulphasalazine, a drug reported as early as 1942 to have therapeutic effects in UC patients [2]. Subsequently, several other formulations of 5-ASA without the sulpha moiety have been developed to minimize possible side effects and provide therapeutic effects. Different preparations of 5-ASA, such as controlled release [3], pH-sensitive, polymer-coated [4] mesalamine formulations, or olsalazine containing two 5-ASA molecules without the sulpha moiety [5], have all been shown to maintain remission significantly better than placebo in 6–12-month, double-blind, placebo-controlled studies in quiescent UC patients. Most studies investigating the efficacy of 5-ASA as a maintenance therapy were only 6 or 12 months in duration—too short to test whether the effect wanes by time. Nevertheless, one study following 150 UC patients treated with fixed doses of oral mesalamine until the appearance of the first relapse found that, aside from 19 dropouts, all 150 patients had relapsed within 9 years [6]. The peak period of relapse appeared between 2 and 3 years, and none of the followed patients developed cancer.

The efficacy of 5-ASA to induce or maintain remission in CD has conflicting results. Indeed, a systematic review has found no evidence showing that 5-ASA preparations are superior to placebo for the maintenance of medically induced remission in patients with CD [7]. According to the latest guideline of the European Crohn's and Colitis Organization (ECCO), 5-ASA preparations are not recommended for maintenance of medically induced remission in CD [8].

### 2.2. Withdrawal

Specific data on the withdrawal of 5-ASA in UC patients in remission is scarce. In 1973 two similar double-blind withdrawal studies were performed with an opposite outcome [9, 10]. Study details can be seen in Table 1. As there was no significant difference between the relapse rates of patients who were switched to placebo and who continued on sulphasalazine, the conclusion of the first study was that it is safe to stop sulphasalazine maintenance therapy in patients who are symptom-free for at least 12 months [9]. To the contrary, as the relapse rates were four times higher in the placebo group, the authors of the second study concluded that salazopyrine maintenance therapy should be continued in UC patients on remission unless there are harmful side effects [10]. The differences between the results may lie in the fact that, in the former study, inclusion and the definition of relapse were based exclusively on symptoms, while in the latter, sigmoidoscopy and rectal biopsy were also performed in a blind manner. Furthermore, the low relapse rate in the treatment group in the second study may be attributed to the high compliance of patients, confirmed by a blood test at 3 months [10].

As the usefulness of maintenance therapy in ulcerative colitis was still unclear, Ardizzone and colleagues performed a randomized, double-blind withdrawal study with 5-ASA (Asacol, 1.2 g/d) to clarify this point in 1999 (Table 1) [11]. Patients were allocated to two groups according to the length of previous remission. Contrary to Group A (remission: 1-2 years), in the group in remission for over 24 months (Group B), the withdrawal of 5-ASA did not influence the relapse rates. Nevertheless, the authors admit that their results must be treated with caution: the sample size was small and patients in Group B were significantly older with longer disease duration than those in Group A. This may explain the results in Group B, as older UC patients and patients with longer disease duration tend to relapse less frequently [33]; therefore the lack of benefit of 5-ASA in this group compared to placebo may reflect the natural history of the disease. The cautious conclusion of this study was that 5-ASA prophylaxis is necessary in patients who are in remission for less than 2 years but questioned whether patients in prolonged clinical, endoscopic, and histological remission with a very low risk of relapse should be treated indefinitely. Nearly ten years later two of the authors have stated that although their results may suggest that a special subgroup of patients could discontinue 5-ASA treatment, the potential effect of 5-ASA to reduce cancer risk still makes long term treatment necessary [34].

### 2.3. Intermittent Therapy

Whether continuous therapy can be replaced by intermittent treatment is also debatable. Fifty UC patients in remission for at least 1 month were enrolled in a 12-month clinical trial performed in Italy [35]. Half of the patients received 5-ASA daily p.o., while the rest of them received 5-ASA treatments only in the first week of each month. Interestingly, the relapse rates were similar in the two groups at the end of the 12-month period. This outcome agreed with previous preliminary results from Dickinson and colleagues, who followed 28 UC patients in remission for 12 months and found that “on-demand” sulphasalazine therapy was equally effective as continuous treatment to prevent relapse [36]. Some authors claim that the lack of benefit from continuous 5-ASA treatment compared to intermittent treatment may come from the low adherence of UC patients to long term treatment [37]. Indeed, these authors speculate that even patients who should be taking 5-ASA on a regular basis tend to use it “on-demand”; that is, they forget their medication until their symptoms remind them to take it again. Therefore the best way to decrease relapse rate would be to find the key to patient adherence to drugs, such as through better patient education.

### 2.4. Possible Side Effects

Early papers have already described side effects of sulphasalazine, the most frequent being gastrointestinal symptoms such as nausea, vomiting, or epigastric discomfort, which affect up to 45% percent of patients [2, 3]. Contrary to these symptoms which are signs of a dose-related intolerance, non-dose-related idiosyncratic reactions also exist [38]. These include hypersensitivity rashes, diarrhea, haematological complications, such as haemolytic anemia or agranulocytosis, and pulmonary and hepatic dysfunction [38–40]. However, 80% of patients intolerant to sulphasalazine are able to tolerate modern 5-ASA preparates [38]. Common side effects of the latter are similar to that of sulphasalazine, such as nausea, vomiting, abdominal pain, headache, and rash [41], but the frequency of these adverse events is comparable to that of placebo [42]. Severe but very rare adverse events may manifest in acute pancreatitis, interstitial nephritis, liver, or pulmonary injury [43]. A recent phase 3, multicenter, 24-month, open-label study focused on side effects in patients in remission from mild to moderate UC, taking mesalamine granules 1.5 g once daily [44]. The authors reported nasopharyngitis to be the most frequent adverse event (13.9%), followed by headache (11.6%) and diarrhea (10.8%). Pancreatic, renal, and hepatic adverse events occurred in less than 6% of patients. Side effects in the above mentioned studies can be seen in Table 2.

## 3. Thiopurines

### 3.1. Efficacy of Maintenance Therapy in the Long Term

6-Mercaptopurine and azathioprine are antimetabolite prodrugs, belonging to the group of thiopurines. It has been discovered early that these drugs work with a time lag of several months in IBD patients [45], and their use to induce remission in CD and UC are controversial [8, 46]. However, the capacity of these drugs to maintain remission in the long term has been proven by single studies and meta-analyses in both CD [47, 48] and UC [49, 50]. Furthermore, due to their steroid sparing effect, they are beneficial for steroid-dependent and steroid-refractory patients [48, 51]. Concerning the efficacy in the long term, a retrospective study conducted in Oxford over a 30-year time period evaluated altogether 622 IBD patients treated with azathioprine [26]. The remission rates were 45% for CD and 58% for UC. At 12, 24, 36, 48, and 60 months the proportion of patients still in remission was 0.95, 0.90, 0.69, 0.63, and 0.62, respectively, and no difference was found between CD and UC patients in relapse rates. The authors have concluded that azathioprine is effective as a long-term maintenance therapy, and the effect does not “wear off” for up to 5 years.

### 3.2. Withdrawal

Compared to aminosalicylates, more attempts have been made to clarify if thiopurines can be withdrawn in UC and CD patients in remission, mostly because the fear from their possible serious side effects makes physicians reluctant to prescribe these medications in the long term.

#### 3.2.1. UC

The only placebo-controlled, double-blind withdrawal trial on UC patients was conducted in 1992 (Table 1) [12]. Patients were taking azathioprine for 6 months or more and were allocated to two groups according to their disease status, namely, in full remission for two months or more without corticosteroids or having a chronic stable disease. The latter was defined as having low-grade symptoms or symptom control with low doses of corticosteroids. The results clearly showed that azathioprine was effective in the maintenance of remission in quiescent UC patients, as withdrawal resulted in twice as many relapses. Patients in a longer remission (at least 6 months, with a median of 12 months) also benefit from continuing maintenance therapy. Data also has shown that, for those patients, who did not achieve remission with azathioprine treatment within 6 months, there is probably no benefit in taking it.

The further available studies assessing the effect of thiopurine withdrawal in UC were retrospective, evaluating the outcome in patients, who stopped thiopurine treatment while in remission (Table 3). A study reviewing charts of patients treated between 1973 and 1992 with 6-mercaptopurine has shown a very high relapse rate after the withdrawal of the drug, and the authors have suggested a long term treatment in those UC patients who respond to therapy [25].

The previously mentioned study from Oxford evaluated not only the long term efficacy but also the relapse rates in 222 IBD (143 UC and 79 CD) patients after stopping azathioprine treatment while being in remission (Table 2) [26]. No statistical difference was observed between UC and CD patients concerning relapse rates, but relapse rates were much higher in patients who stopped azathioprine compared to those who continued it. The authors have concluded that azathioprine is effective with a low toxicity in IBD patients for up to 5 years. However, they cautiously refrained from therapeutic suggestions.

In a study conducted in Italy, relapse rates after azathioprine withdrawal in steroid-free remission were high, similarly to previous studies (Table 2) [27]. In this study, several predictive factors of relapse after azathioprine withdrawal have been identified, which will be detailed later. In 10% of cases, the outcome was colectomy, which could be predicted by drug-related toxicity as the cause of azathioprine withdrawal, no post-azathioprine drug therapy, and treatment duration.

#### 3.2.2. CD

In CD, the picture is more complex. An early double-blind withdrawal trial showed the efficacy of azathioprine maintenance therapy compared to placebo, irrespective of complementer anti-inflammatory drug use (Table 1) [13]. One patient developed pancytopenia and died despite of the withdrawal of azathioprine. This patient had been taking azathioprine for more than ten years before entering the trial, where he continued with the azathioprine treatment. These results suggested that azathioprine is efficient in the maintenance of remission in the long term but shed light also on the possible fatal side effects, and the optimal duration of therapy was not yet determined.

The efficacy of thiopurines in the long term was also confirmed by a follow-up study on 120 CD patients (Table 3) [28]. The median length of remission in patients who continued on 6-mercaptopurine was 32 months, while it was only 16 months in patients who stopped treatment. The authors concluded that 6-mercaptopurine should be continued indefinitely, if the drug is well tolerated.

A retrospective study in France on CD patients showed no further benefit during and after the fifth year of remission in patients who continued to take azathioprine or 6-mercaptopurine compared to those who had stopped it (Table 3) [16]. This study was the first to raise the suggestion that thiopurines may be discontinued in CD patients who are in remission for at least 4 years.

A small randomized open withdrawal trial on CD patients in stable remission without steroids have confirmed the efficacy and need to continue azathioprine at least for 3 years, but did not give information on the previously questioned efficacy beyond 4 years (Table 1) [14].

A multicenter European retrospective study on UC and CD patients has shown that, in the first 4 years, thiopurines were equally efficient in the two diseases (Table 3) [29]. Long term therapy beyond 4 years had a significant beneficial effect on disease activity in CD patients and reduced steroid requirement in both CD and UC. Nevertheless, in CD patients in steroid-free remission, no relapse was seen after the discontinuation of the drug after 3-4 years. Therefore it has been concluded that discontinuation of thiopurine therapy may be considered in this specific group of patients. However, in other CD and UC patients the continuation of treatment is recommended.

So far, the opinion that thiopurine therapy may lose its benefit after 4 years of treatment in CD patients was based only on retrospective results [16, 29] and later has been challenged by several authors. Indeed, inspired by their previous results, the same French group conducted a randomized, double-blind, controlled noninferiority withdrawal trial to confirm their observation that CD patients in remission on azathioprine for more than 3.5 years are at low risk of relapse when azathioprine is discontinued (Table 1) [15]. Although included patients were steroid-free for at least 42 months, azathioprine continuation was found superior to azathioprine withdrawal, and the authors concluded that the prolonged treatment with azathioprine should be continued beyond 3.5 years.

The extension of this study was published four years later [18]. All patients in remission, who had stopped azathioprine following randomization in placebo arm in the previous trial (43 patients) and those who had discontinued azathioprine after the end of that trial (23 patients) were included. The median followup was 54.5 months. Out of the 66 patients, 32 (48.4%) relapsed during follow up. The cumulative probabilities of relapse at 1 year and 3 and 5 years were 14%, 52.8%, and 62.7%, respectively. Time to relapse curves of those patients, who had stopped azathioprine due to randomization in placebo group (median treatment duration: 62 months) were not different from those who stopped treatment after the end of the trial (median treatment duration: 80 months).

A further study aimed to assess, whether the effect of azathioprine wears off after 4 years in CD patients [17]. Namely, one hundred steroid-dependent CD patients were allocated to two groups, depending on the previous duration of remission with azathioprine treatment and followed for one year. Steroids and 5-ASA were tapered off before enrollment. Relapse rate was 19.6% in the group of 58 patients in remission for 2–4 years and 11.9% in the group of 42 patients in remission for more than 4 years. This study has demonstrated that the effect of azathioprine does not wane after 4 years of therapy.

It is interesting to note that none of the above-mentioned studies have evaluated 6-thioguanine levels at the time of therapy withdrawal and the risk of relapse. 6-thioguanine nucleotides are the active metabolites of azathioprine, and their higher level in red blood cells in CD and UC patients is associated with an increased rate of steroid-free remission [52].

### 3.3. Possible Side Effects

5 to 10% of patients do not tolerate thiopurines due to idiosyncratic adverse events, most commonly fever, nausea, diarrhea, rash, abdominal pain, pancreatitis, and allergic reactions that mostly occur within the first 2-3 weeks of therapy [53, 54]. Myelosuppression and consequent leucopenia and/or thrombocytopenia are the most common and potentially lethal hematological complications, which develop in 2.2 to 15% of patients [53, 55]. The onset of myelosuppression can range from 2 weeks to 11 years from the initiation of therapy and is usually reversed by dose reduction, but severe opportunistic infections may potentially occur [55, 56]. A mild elevation in liver function tests is not uncommon and usually responds to dose reduction [53]. Less frequently severe hepatotoxicity, for example, nodular regenerative hyperplasia, may develop, leading to progressive liver damage and portal hypertension. The most delicate topic regarding long term consequences of thiopurine therapy is the incidence of cancer and lymphomas. Indeed, patients under thiopurine therapy are exposed to an increased risk of nonmelanoma skin cancer; therefore they should use protection against UV radiation and undergo lifelong regular dermatologic checkups [53, 57, 58]. Regarding lymphomas, a meta-analysis has found a fourfold risk in IBD patients on thiopurine treatment [59], while a large prospective study has identified a 5.28 hazard ratio in IBD patients taking thiopurines [60]. Whether the increased risk is related to the drug or to the disease itself is not known, but discontinuation of therapy can decrease the risk of lymphomas [61]. Young, smoking females, but not nonsmokers, are at increased risk of cervical dysplasia [62]. Still, other authors have observed no increased risk of malignancy in IBD patients on thiopurine treatment [63, 64]. Side effects in the above-mentioned studies are shown in Table 2.

## 4. Methotrexate

### 4.1. Efficacy of Maintenance Therapy

Methotrexate is generally reserved for CD patients with active or relapsing disease, who are refractory to or intolerant of thiopurines or anti-TNF agents [8]. A recent meta-analysis has concluded that methotrexate at a dose of 15 mg/week by intramuscular route is superior to placebo for maintenance of remission in CD for up to 40 weeks, while data on oral route are insufficient for a clear conclusion [65]. In the case of CD patients on methotrexate maintenance therapy, the probability of remaining in remission ranged from 71 to 90% at one year, 59 to 73% at two years, and 51 to 52% at three years [19, 66].

Although some studies have demonstrated beneficial effects of methotrexate in the maintenance of remission in UC, contradictory results also exist [19]. Different doses and the route of administration highly affect the outcome; intramuscular route has been found the most effective. A study from a Spanish group found that 90%, 80%, and 50% of UC patients on methotrexate therapy were still on remission at 1 year and 2 and 3 years, respectively [67]. The ECCO consensus stated that the evidence is currently insufficient to recommend methotrexate for UC [8].

### 4.2. Withdrawal

There is very little data on discontinuation of methotrexate therapy in IBD patients on remission, as specific withdrawal studies are missing. A previously mentioned retrospective study reported an extremely high relapse rate after discontinuation of therapy (Table 3) [19].

### 4.3. Possible Side Effects

Safety concerns of methotrexate have a large impact on its limited use in IBD patients. Studies report the incidence of side effects rising up to 22%, which lead to a discontinuation of treatment in 10–18% of cases [67]. Short term side effects are mainly gastrointestinal, such as nausea, vomiting, diarrhea, and stomatitis, which can mostly be prevented by the coadministration of folic acid two or three days apart from methotrexate [8]. In the long term, bone marrow toxicity, hepatotoxicity, and pneumonitis can occur. The occurrence of bone marrow toxicity is low in patients with normal renal function and it can be further reduced by coadministration of folic acid [68]. A meta-analysis of clinical trials has found a low occurrence of hepatotoxicity (defined as up to a 2-fold increase over the upper limit of the normal hepatic aminotransferase levels) in IBD patients treated with methotrexate, with a rate of 0.9 per 100 person-months [69]. In children, abnormal liver biochemistry was detected in 10.2% of cases, dose reductions were required in 6.4%, while in 4.5% of cases methotrexate had to be completely stopped, according to a large meta-analysis [70]. Therefore risk factors of hepatotoxicity, for example, alcohol abuse, chronic viral infections, diabetes, and obesity, must be ruled out before the initiation of therapy, and liver monitoring during the course of treatment is extremely important [67]. The prevalence of pneumonitis is reported to be 0.3–7.5% in rheumatoid arthritis patients [71], but no cases were reported in large series [72]. Side effects in the above-mentioned retrospective study from Fraser are detailed in Table 2. According to new expert opinions, the side effect profile of methotrexate is not worse than that of azathioprine. Therefore a more widespread use in IBD, similarly to rheumatologic diseases, can be expected in the future.

## 5. Biological Therapies

### 5.1. Efficacy of Maintenance Therapy

Infliximab, a chimeric monoclonal antibody against tumor necrosis factor alpha (TNF-*α*), and adalimumab, a fully human monoclonal IgG1 anti-TNF-*α* antibody, were both found effective as a maintenance therapy in UC and CD [73–77]. Regarding the effects in the long term, in a group of moderate-to-severe, therapy refractory UC patients, 67% have achieved response with infliximab therapy, and out of these patients, 68% had a sustained response during a median followup of 33 months [73]. In CD patients treated with infliximab, sustained clinical benefit was observed in 63.4% of patients up to a median followup of 55 months, while 68.3% of patients were still on infliximab therapy [21]. Furthermore, in a retrospective study, in CD patients with complete response on infliximab the cumulative probability of being free of relapse was 97.2% at 12 months, 90.3% at 24 months, 81.7% at 36 months, 73.5% at 48 months, and 61.3% at 51 months [78]. From this data it can be concluded that infliximab is effective in CD patients in the long term, but, as a systematic review has pointed out, the annual risk for loss of infliximab response is around 13% per patient-year, which requires therapy intensification [79]. Adalimumab has been found significantly more effective compared to placebo for the maintenance of remission in UC and CD patients for up to one year [75–77]. A 4-year followup of adalimumab treated UC patients has recently reported that remission, mucosal healing, and improved quality of life is well maintained by adalimumab up to the 4th year of treatment [80].

### 5.2. Withdrawal

#### 5.2.1. UC

In a Danish observational, retrospective study, out of 97 UC patients, 28 (30%) have stopped infliximab therapy due to a stable steroid-free remission, according to their treating physicians' global judgment (Table 3) [30]. 25% of patients relapsed within one year, and no factors could be identified that correlated with relapse or with prolonged remission (remission > 1 year). Concomitant immunosuppression did not influence the relapse rate.

According to the Hungarian National Health Insurance Fund Administration reimbursement regulations, biological therapy must be discontinued after a 1-year treatment period in UC and CD patients who achieved remission. These regulations have created the basis to observe the effects of infliximab and adalimumab withdrawal in IBD patients [23, 24]. In a recent prospective observational study, UC patients, who have achieved clinical remission after a 1-year period of infliximab therapy, were followed after the discontinuation of treatment (Table 3) [24]. 35% of patients relapsed within one year after the withdrawal of infliximab therapy.

#### 5.2.2. CD

A study conducted in Spain has collected clinical data from three centers of the outcome after discontinuation of infliximab therapy in CD patients (Table 3) [20]. The cumulative probability of being relapse-free was 45% and 34% at 6 and 12 months in case of perianal disease, while in case of luminal CD it was 83% at 12 months. Perianal disease was the only factor predicting relapse. The authors suggest that infliximab discontinuation is not recommended in perianal CD, as early relapse is extremely common.

In a large observational study evaluating the long term effects of infliximab therapy in CD, treatment was stopped in 110 patients in remission (Table 3) [21]. The authors have found that one in three patients can stop infliximab therapy and still stay in remission while continuing on immunosuppressants, but no factor could be identified which would predict a favourable outcome after drug withdrawal. In a longitudinal cohort study conducted in Canada, data of CD patients who had discontinued infliximab treatment while in infliximab-induced steroid-free remission was evaluated (Table 3) [32]. Similarly as in the previous study, 35% of patients remained in sustained remission after infliximab withdrawal, but the authors were unable to find a factor responsible for this outcome. In the previously mentioned Danish retrospective study, 53 of 219 CD patients (24%) discontinued infliximab therapy due to clinical remission (Table 3) [30]. Around 40% of these patients could benefit from a sustained remission. Those CD patients who relapsed had significantly longer disease duration, but no factor was associated with a prolonged remission.

In a multicenter prospective study called STORI, 115 CD patients were included, who were treated for at least 1 year with infliximab and an antimetabolite (methotrexate/azathioprine/6-mercaptopurine), and have been in corticosteroid-free remission for at least 6 months before inclusion (Table 3) [22]. The relapse rate was nearly 44% after 1 year of infliximab withdrawal. Several individual risk factors for relapse were identified in this study, which will be detailed later. The authors suggest that by assessing these parameters, patients with low risk of relapse can be selected and in these patients, infliximab can be stopped. Others have claimed that the elevated CDEIS score, increased CRP and fecal calprotectin levels show that, in the case of these patients, the disease was not under “tight control,” and in the era of mucosal healing, mucosal flair would better represent the outcome than clinical relapse [81].

A multicenter, prospective observational study called RASH followed 121 CD patients who were in clinical remission after 1 year therapy with infliximab (*n* = 87) or adalimumab (*n* = 34) (Table 3) [23]. Similarly to the previous study, one-year relapse rate was 45%, and many risk factors predicting a relapse after withdrawal have been identified. A huge difference between the patient population of the RASH study and the STORI study was that, in the former, biological treatments were stopped even if steroids could not be tapered off, while, in the latter, patients were in a corticosteroid-free remission 6 months before inclusion.

### 5.3. Possible Side Effects

Although infliximab and adalimumab are considered safe in the short term, many concerns exist regarding their long term use. A large case-control study with a mean followup of 1.9 years has shown no difference between the mortality rate of infliximab-treated and noninfliximab treated CD patients [82]. Similarly, a retrospective cohort study with a median follow-up time of 58 months (of IBD patients) and 144 months (of controls) has found no difference between the mortality rate of infliximab-treated IBD patients and of IBD patients not receiving any biological therapy [83]. However, in this study the death of an elderly patient due to Aspergillus sepsis was directly related to infliximab therapy. Indeed, one of the main concerns is the increased risk of infections. The retrospective study did not find a significant difference between the infection rates of the two groups, but the risk of infections was significantly increased when concomitant corticosteroids were administered [83]. Additionally, two patients on infliximab were diagnosed with extraintestinal tuberculosis, underlining the importance of detecting mycobacterial infection before the start of biological therapy, even though it is known that response to a tuberculin skin test may be anergic in up to 83% of CD patients on immunosuppressive therapy [84]. In the case-control study, infliximab was not identified as an independent predictor of serious infections, contrary to corticosteroid use [82]. Another important concern is the development of malignancies in patients on biological therapies. In placebo-controlled trials, according to a systematic review, the risk of malignancies was not increased in patients treated with anti-TNF-*α* therapy, but the followup was only one year, and therefore no conclusions can be drawn concerning the long term effects [85]. In the previously mentioned retrospective study with a follow-up time of more than 5 years, the risk of malignancies was also not elevated in infliximab-treated IBD patients [83]. At this point, an evidence-based guideline cannot be given, but tight surveillance is advisable to detect precancerous lesions and early cancers in patients receiving biological treatments [51]. A further rare but specific problem associated with the use of anti-TNF-*α* treatment is demyelinating disease, which usually mimics multiple sclerosis [51, 83]. In a retrospective analysis aiming to determine the frequency of this adverse event, during a four-year period of 550 rheumatoid arthritis patients treated with anti-TNF-*α* agents, 6 cases of demyelinating disease were identified [86]. Further, frequent complications associated with the use of infliximab are dermatological symptoms, often diagnosed as psoriasiform dermatitis and eczema [83]. These symptoms can occur in up to 20% of patients, but in most cases respond well to topical steroid treatment and rarely require the discontinuation of therapy. Side effects of the previously detailed withdrawal studies are shown in Table 2.

## 6. Specific Aspects

### 6.1. Length of Previous Remission

It is an important question whether a long remission while on therapy can predict a sustained remission after drug withdrawal. Results are controversial, and many of them point to the direction that the duration of previous remission does not predict the outcome after drug withdrawal (Table 4). Time to relapse and/or the relapse rate after withdrawal were similar in patient groups regardless of the length of previous therapy with the drug or the duration of preceding remission in many studies [9, 10, 13, 18, 26, 28]. In the study showing a better outcome after 5-ASA withdrawal in patients in remission for more than 2 years compared to those in remission for 1-2 years, the patients with longer remission were significantly older, which can explain the observed difference, as lower age has been found a predictive factor of relapse in several studies [11]. In the study with azathioprine withdrawal in UC patients, a significant difference could be found only between the groups of a short treatment duration (3–6 months) and very long treatment duration > 48 months [27]. Altogether the results suggest that, except from a very short treatment time, other factors, for example, the depth of remission, are more important than the duration of remission.

### 6.2. Mucosal Healing

Mucosal healing is defined as the “complete absence of all inflammatory and ulcerative lesions in all segments of gut” at endoscopy [87]. Several different endoscopic scoring systems have been used in clinical trials on UC patients to assess endoscopic activity, the Mayo endoscopic score being the most popular. Recently mucosal healing is often used as an important endpoint to assess the therapeutic effect in UC and CD. It is associated with a lower rate of hospitalisation and surgery, but its predictive role of a sustained remission after drug withdrawal is not yet clear.

As mucosal healing is a novel entity, its connection with a prolonged remission in earlier drug withdrawal studies could not been tested. In the study of Dissanayake and Truelove a sigmoidoscopy with rectal biopsy was performed at enrollment in the study, in case of a suspected relapse and at the end of the 6-month study period [10]. Only patients with a normal sigmoidoscopic finding and a rectal biopsy without significant inflammation entered the withdrawal study. Similarly, in the study of Ardizzone, only patients with no signs of active inflammation on sigmoidoscopy and with a stable histological remission, defined as grade 0 (absence of neutrophils) according to the criteria of Truelove and Richards, were selected for the study [11]. Due to this design no information is available on the association between endoscopic remission and the rate of relapse after 5-ASA discontinuation.

Similarly, in case of thiopurines, no studies were designed specifically to answer if there is a link between the endoscopic-histological picture at therapy withdrawal and the risk of relapse. In the study of Hawthorne et al. a sigmoidoscopy was performed at the inclusion, and only patients with a mucosal appearance of grade 0 or 1 (Baron, 0: normal mucosa; 1: granular or oedematous mucosa with loss of vascular pattern) were eligible for the study [12]. However, no data is available concerning the difference of the relapse rate between patients with grade 0 or grade 1 mucosal appearance. In case of the study of Lémann and colleagues, out of the 83 enrolled patients, 45 had a colonoscopy at baseline, and only 36% of them had complete mucosal healing [15]. The authors state that endoscopic lesions or ulcerations were not predictive of relapse, but data on the risk of relapse in patients with or without mucosal healing are not shown.

Concerning biological therapy, more information is available on the topic. Indeed, in UC patients, no significant correlation could be seen between mucosal healing at the end of infliximab therapy and the outcome of withdrawal [24]. Similarly, in the RASH study, no correlation was found between mucosal healing and the need to restart biological therapy in CD patients, although the number of patients with endoscopic evaluation was limited [23]. Furthermore, in a recent prospective study designed with the aim to answer this question, mucosal healing could not predict the outcome of anti-TNF-*α* therapy withdrawal in 41 CD and 22 UC patients (Table 3) [31]. In this study, all patients received either infliximab or adalimumab treatment for 12 months before withdrawal and were followed for one year. At the end of the treatment period, mucosal healing was achieved in 56% of CD and 32% of UC patients, while deep remission (mucosal healing + clinical remission) in 54% of CD and 23% of UC patients. Interestingly, 59% of CD and 100% of UC patients in deep remission had to be retreated within one year, while out of the total study population, 78% of CD and 59% of UC patients. These results suggest that evaluation of mucosal healing should not replace the assessment of clinical activity and the patient's general condition, when considering therapy withdrawal.

The use of a new treatment target, the so-called histological remission, is emerging in IBD [88], which may appear as a novel endpoint in withdrawal studies in the future. However, at present numerous histological scoring systems exist in IBD, without being properly validated. The role of histological remission to predict complications in IBD is not well established, and it is not yet clear if this new term can be used as an endpoint in the treatment of IBD.

### 6.3. Chemoprevention

The increased risk of colorectal cancer (CRC) in UC has long been recognized. An early study performed on UC patients, who grew up in the era when effective treatment for IBD hardly existed, found a 20% risk of colon cancer per decade in children at risk, starting after 10 years of disease onset [89]. It has since been observed that the risk of CRC is positively correlated with the duration and the anatomic extent of the colitis, the degree of inflammation, the presence of primer sclerotizing cholangitis, and a positive family history for CRC [90]. In case of CD patients with colonic involvement, the risk of CRC is similarly elevated to UC patients, while in CD patients without colonic involvement it is comparable to the general population [91]. In case of small bowel involvement, the risk of small bowel adenocarcinoma is extremely elevated in CD patients compared to the general population. However, the overall incidence of disease is low. In the last decades, a gradual reduction of CRC risk has been observed in IBD patients, likely due to the effective medical and surgical treatments [90]. Indeed, population-based studies from the last decade have found no increased overall risk of CRC in UC patients compared to the general population, with a cumulative risk of only 2% in 30 years [92, 93]. Still, in UC patients with an extensive disease, a slight increase in CRC risk could be observed [93].

Several data support the hypothesis that the long term use of 5-ASA decreases the risk of CRC in UC, although the data are heterogeneous due to methodological differences [94]. The ECCO recommends the prolonged use of 5-ASA to prevent CRC [51]. 5-ASA prevents CRC by interfering with various mechanisms in CRC cell biology apart from simply controlling inflammation [95]. Nevertheless, as severe inflammation is also an independent risk factor for the development of CRC in UC, theoretically any treatment that reduces the inflammation can have chemopreventive effects. A recent study has found that histological severe inflammation increases the odds ratio of CRC to more than 30 in UC patients, whereas the use of 5-ASA or thiopurines markedly decreased the risk of CRC [96]. Furthermore, a case-control study has also demonstrated that treatment with azathioprine, 6-mercaptopurine, or methotrexate all reduced CRC risk in UC patients [97]. Anti-TNF-*α* therapy has also been found protective against CRC [98]. Altogether it seems that any therapy able to induce a complete mucosal healing is chemoprotective.

## 7. When to Stop?

It is well known from the clinical practice that the compliance of patients is rapidly decreasing after they have achieved remission, and if physicians are reluctant to reduce the number of tablets, the patients tend to do it themselves. Therefore it is essential to identify those patient groups where the maintenance therapy can be discontinued without a high risk of relapse.

In case of 5-aminosalicylic compounds, many authors are unanimous in the opinion that 5-ASA should be continued lifelong in UC patients in remission unless side effects are present [10, 34]. Nevertheless, it is possible that intermittent 5-ASA therapy, which is associated with lower costs, can be as effective as continuous therapy [35, 36] and can possibly be favored by patients, but it should be remembered, that it is not known if intermittent administration of 5-ASA is still efficient for chemoprevention.

Regarding methotrexate, further studies on large number of patients are needed to determine the optimal therapy duration in IBD, but it seems that relapse rates after therapy withdrawal are outstandingly high.

In case of azathioprine, for those patients who did not achieve remission within 6 months, there is probably no benefit in taking it [12]. However, among those patients who have reached remission with thiopurines or anti-TNF-therapies several risk factors predisposing to a relapse after drug withdrawal have been identified, which are detailed in Table 5.

**Figure 1:**
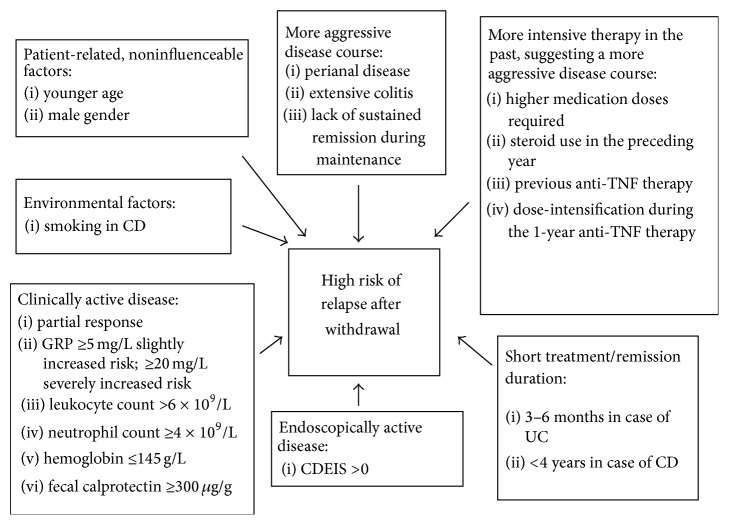
Risk factors predisposing to a relapse after drug withdrawal.

These factors can be grouped in different categories shown in Figure 1, and should be assessed in case of a planned therapy withdrawal. The absence of surgical resection as a risk factor does not fit into this picture, as surgical resection is known to be associated with a more aggressive disease course [22, 99]. The most important message for the clinician is that the discontinuation of therapy should be considered only in those patients, who have a milder disease course and who are in a complete remission, with no alterations in laboratory parameters and with a negative colonoscopy. Nevertheless, mucosal healing should not be a more important endpoint than the patients' global well-being and clinical parameters [31]. As seen in Table 5 and Figure 1, younger and male patients are at increased risk of relapse, similarly to patients who have required a more intensive therapy in the past.

The ECCO states that, in case of CD patients in remission on azathioprine, “cessation may be considered after four years of remission. Benefit and risks of continuing azathioprine should be discussed with individual patients.” [8] In the study of Treton and colleagues, in a special subgroup of CD patients in steroid-free remission, who are free from certain risk factors, such as high CRP level and neutrophil count, low hemoglobin level, and non-smoking status, no patients relapsed within 18 months, suggesting that in this subgroup the treatment can be suspended for a limited time period, if necessary [18]. This could be an option in case of a planned pregnancy or breastfeeding, although smoking must be stopped.

Considering biological treatment, financial factors often influence the decision on the continuation or withdrawal of therapy. The introduction of biosimilars in the market will possibly reduce the present high costs and this may also be reflected in therapeutic decisions. Until clear guidelines for the optimal duration of therapy can be formulated, the opinions of physicians remain quite discrepant. In a questionnaire filled out by Canadian gastroenterologists, 77% would prefer to continue infliximab treatment indefinitely, if it is well tolerated and effective, while 12% opted to discontinue it after 1 year, and 2% would use it for a maximum of 6 months [100]. One must also keep in mind that similar to steroid-dependency, dependency from other treatments, for example, anti-TNF therapy, can develop in IBD patients [101]. In the upcoming era of personalized medicine, in the case of CD patients, where the role of genetic factors in the pathogenesis is well known, it would be interesting to identify a specific mutation that predicts a quick relapse after the withdrawal of therapy. However, a recent study could not identify any IBD5 and NOD2/CARD15 polymorphisms predictive of the outcome after withdrawal of infliximab therapy [102].

## 8. Retreatment after Relapse

After a relapse following a therapy withdrawal, the required treatment highly depends on the reason of the discontinuation of the drug. If the drug was discontinued due to side effects or intolerance, then new therapeutic solutions have to be found. Nevertheless, if the drug was well tolerated, the most obvious solution is the reintroduction of the previously efficacious therapy. Results from studies on azathioprine or anti-TNF medications show mostly excellent results with reintroduction of therapy. In the study of Treton and colleagues, 23 of the 32 relapsed patients were retreated with azathioprine alone or in combination with steroids, and among them, 22 have successfully achieved remission [18]. The only patient who did not respond to azathioprine alone was suffering dominantly from anoperineal symptoms. One patient received a combination of azathioprine and infliximab. The further 8 patients were not retreated with azathioprine, but four of them were operated for intestinal stenosis, one was treated with infliximab and three with methotrexate.

In case of anti-TNF treatment, many data are available concerning retreatment after withdrawal. In the study of Kim et al., out of 36 relapsing CD patients, 25 were retreated with infliximab, and 24 patients (96%) reached complete clinical remission [28]. One patient among the 25 suffered an acute severe infusion reaction at the second infliximab infusion of the new series, which led to the discontinuation of infliximab. In the same study, 7 out of 10 relapsing UC patients were retreated with infliximab, which resulted in a complete clinical remission in 5 patients, partial remission in one patient, and no effect in one patient, requiring colectomy. In the Hungarian study on UC patients, infliximab treatment had to be restarted in 35% of patients, which resulted in a 94% remission, while 6% needed surgery [24]. In the STORI study, of those 40 patients, who were adequately assessed 30 days after infliximab retreatment, 93% were in remission and 98% had a clinical response [22]. In this study, available serum samples were tested for anti-infliximab antibody. Before retreatment with infliximab, the 39 available serum samples were found negative, while among the 41 available serum samples before the second retreatment infusion, 11 were negative and 30 inconclusive. No infusion reaction or significant delayed reaction has occurred in the retreated patients up to the third retreatment. In the RASH study, 45% of CD patients required retreatment with anti-TNF-*α* within one year, which has successfully induced clinical remission in 54.7% of patients, while 9.1% underwent surgery [23]. In case of reinitiation of therapy, 4% of patients suffered from mild side effects and 6% had an infusion reaction.

## 9. Conclusions

Knowledge on the outcome after withdrawal of different medical therapies in IBD patients who have successfully achieved remission is constantly growing but is still insufficient to create universal guidelines. As a final conclusion, the relapse rates after withdrawal of a well-established therapy in IBD patients are generally high, but in case of specific patients, the discontinuation of therapy can be considered. This decision must be made on an individual basis by the help of the predictive risk factors detailed above and must be discussed with the individual patient, after providing all the necessary information on the risks of the cessation and the continuation of therapy. More, well-designed prospective studies are needed to answer all the remaining questions concerning the optimal duration of therapy in IBD patients in remission.

## Figures and Tables

**Table 1:** Drug withdrawal studies.

Authors	Year	Disease	Treatment	Number of patients	Length of preceding remission	Number of patients allocated to different groups	Study duration	Outcome
Riis et al. [9]	1973	UC	Sulphasalazine	49	≥12 months	Continue on drug: 25, placebo: 24	6 months	No significant difference between the relapse rates (24 versus 29%)

Dissanayake and Truelove [10]	1973	UC	Sulphasalazine	64	≥12 months	Continue on drug: 33, placebo: 31	6 months	Significantly higher relapse rate in the placebo group (>50% versus 12%)

Ardizzone et al. [11]	1999	UC	5-ASA	112	≥12 months	Group A (in remission for 1-2 years): continue on drug: 26, placebo: 35; Group B (in remission for >2 years): continue on drug: 28, placebo: 23	12 months	Group A: no significant difference after 6 months, but significantly higher relapse rates in the placebo group after 12 months (23% versus 49%). Group B: no significant difference at 6 and 12 months.

Hawthorne et al. [12]	1992	UC	Azathioprine	79	≥2 months or chronic stable disease	Remission group: continue on drug: 33, placebo: 34; chronic stable disease group: continue on drug: 7, placebo: 5	12 months	Remission group: significantly higher relapse rate in the placebo group (59% versus 36%); chronic stable disease: azathioprine group: 71% relapsed within 6 months; placebo-treated patients: 40% relapsed

O'Donoghue et al. [13]	1978	CD	Azathioprine	51	≥6 months	Continue on drug: 24, placebo: 27	12 months	Significantly higher relapse rate in the placebo group at 6 months (25 versus 0%) and 12 months (41 versus 5%)

Vilien et al. [14]	2004	CD	Azathioprine	28	≥3 months (≥2 years treated with azathioprine)	Continue on drug: 13, stop drug: 15	12 months	Significantly higher relapse rate in the no azathioprine group at 12 months (53 versus 15%), higher azathioprine dose-subgroup (>1.60 mg/kg/day: 67 versus 11%)

Lémann et al. [15]	2005	CD	Azathioprine	83	≥42 months	Continue on drug: 40, placebo: 43	18 months	Significantly higher relapse rate in the placebo group (20.9 versus 7.5%)

**Table 2:** Adverse events in pro- and retrospective studies.

Author	Year	Drug	Adverse events/side effects (number of cases)	Death from (number of cases)
Dissanayake and Truelove [10]	1973	Sulphasalazine	Nausea (2); headache (1)	0

Ardizzone et al. [11]	1999	5-ASA	Abdominal pain, bloating, diarrhea (3)	0

O'Donoghue et al. [13]	1978	Azathioprine		Pancytopenia (1)

Hawthorne et al. [12]	1992	Azathioprine	Transient bone marrow suppression (2)	0

Bouhnik et al. [16]	1996	Azathioprine/6-mercaptopurine	Leucopenia (18), thrombocytopenia (2), liver abnormalities (4), urinary tract infections (4), malignant melanoma (1), cutaneous basal cell carcinoma (1), renal carcinoma (1), and brain lymphoma (1)	0

Lémann et al. [15]	2005	Azathioprine	Mild leucopenia reversed with dose reduction (1)	Myelodysplastic syndrome (1)

Mantzaris et al. [17]	2007	Azathioprine	Headache (17), paraesthesias (1), flu (7), herpes simplex (5), herpes zoster (2), bronchitis (2), transient psoriasis rash (1), transient leucopenia (3), significant leucopenia (2), no malignancies	0

Treton et al. [18]	2009	Azathioprine	Moderate leucopenia, reversed by dose adjustment (3)	Breast cancer (1); disseminated varicella (1 treated with azathioprine in combination with infliximab)

Fraser et al. [19]	2002	Methotrexate	Nausea and vomiting (7), increased diarrhea (2), severe stomatitis (1), leucopenia (3), pneumonia (1), fever (1), shingles (1), elevated liver enzyme (2), photosensitive rash (1)	Multiple organ failure (1)

Domènech et al. [20]	2005	Infliximab	Acute infusion reactions (5); no malignancies, opportunistic infections	0

Schnitzler et al. [21]	2009	Infliximab	Acute infusion reactions (15), delayed infusion reactions (33), herpes zoster (1), abdominal tuberculosis (1), neuritis optica (1), central demyalinising lesion (1), extensive multiple sclerosis-like neurological symptoms (1)	Aspergillus infection (1)

Louis et al. [22]	2012	Infliximab	No serious adverse events	0

Molnár et al. [23]	2013	Infliximab or adalimumab	In 10.9% of patients, none of them serious	0

Farkas et al. [24]	2013	Infliximab	No serious adverse events	0

**Table 3:** Retrospective and observational studies.

Authors	Year	Study type	Disease	Treatment	Number of patients	Length of preceding therapy	Outcome after withdrawal
George et al. [25]	1996	Retrospective	UC	6-Mercaptopurine	105		Relapse: 87%

Fraser et al. [26]	2002	Retrospective	UC and CD	Azathioprine	222 IBD (143 UC and 79 CD)		Relapse at 1 year: 37%; 2 years: 56%; 3 years: 66%; 4 years: 72%; 5 years: 75%

Cassinotti et al. [27]	2009	Retrospective	UC	Azathioprine	127	Median: 47 months	Relapse at 1 year: 35%, 2 years: 49%, 3 years: 59%, 4 years: 61%, 5 years: 65%

Kim et al. [28]	1999	Retrospective	CD	6-Mercaptopurine	36	≥6 months	Relapse at 1 year: 36% (versus 29% in those on drug), 2 years: 71% (versus 45% in those of drug), 3 years: 85% (versus 55% in those on drug), 5 years: 85% (versus 61% in those on drugs)

Bouhnik et al. [16]	1996	Retrospective	CD	Azathioprine/6-mercaptopurine	42	>6 months	Relapse at 1 year: 38%, 3 years: 61%, 5 years: 75%

Holtmann et al. [29]	2006	Retrospective	UC and CD	Azathioprine/6-mercaptopurine	160 CD and 208 UC	<3 years, 3-4 years or >4 years	Median disease flare/year: in CD: 0 in all duration groups; in UC: <3 years: 0, 3-4 years: 0.48; >4 years: 0.96

Fraser et al. [19]	2002	Retrospective	CD and UC	Methotrexate	19	Mean: 17.1 months	Relapse at 6 months: 58%, 12 months: 79%, 18 months: 84%

Steenholdt et al. [30]	2012	Retrospective	UC	Infliximab	28		Relapse at 1 year: 25%, 10 years: 88%
			CD		53		Relapse at 1 year: 39%, 4.5 years: 60%

Farkas et al. [31]	2014	Prospective observational	UC	Infliximab or adalimumab	22	1 year	Relapse at 1 year: 59%
			CD		41		Relapse at 1 year: 78%

Domènech et al. [20]	2005	Retrospective	CD	Infliximab	23 luminal CD patients receiving only induction therapy (3 infusions)	6 weeks	Relapse at 2 months: 100% in partial remission group; 6 months: 28% in complete remission group
					23 luminal or perianal CD patients receiving infusions every 8 weeks	1 year	Relapse at a median of 8.8 months: 48%

Schnitzler et al. [21]	2009	Prospective observational	CD	Infliximab	110	Median: 6.2 months	Median length of remission: 47.3 months

Waugh et al. [32]	2010	Retrospective	CD	Infliximab	48	Median: 15.6 months	Relapse at a median of 477 days: 50%, at a median of 4.1 years: 65%

Louis et al. [22]	2012	Prospective observational	CD	Infliximab	155	≥1 year	Relapse at 1 year: 43.9%, 2 years: 52.2%

Molnár et al. [23]	2013	Prospective observational	CD	Infliximab or adalimumab	121	1 year	Relapse at 1 year: 45%, 2 years: 88%

Farkas et al. [24]	2013	Prospective observational	UC	Infliximab	51	1 year	Relapse at 1 year: 35%

**Table 4:** The influence of the duration of remission or previous therapy on the outcome after drug withdrawal.

Author	Year	Disease	Drug	Length of previous remission in the different groups	Length of previous therapy with the drug in the different groups	Better outcome at withdrawal after longer remission/therapy
Riis et al. [9]	1973	UC	Sulphasalazine	12–24 months versus >24 months		No

Dissanayake and Truelove [10]	1973	UC	Sulphasalazine	1–3 years versus >3 years		No

Ardizzone et al. [11]	1999	UC	5-ASA	1-2 years versus >2 years		Yes

Hawthorne et al. [12]	1992	UC	Azathioprine	Continuous analysis		Yes; *p* = 0.1

Fraser et al. [26]	2002	UC	Azathioprine		<2 years versus 2–4 years vs. >4 years	No

Cassinotti et al. [27]	2009	UC	Azathioprine		3–6 months versus 48 months	Yes

O'Donoghue et al. [13]	1978	CD	Azathioprine	<1 years versus 1-2 years versus >2 years	<1 years versus 1-2 years versus >2 years	No

Bouhnik et al. [16]	1996	CD	Azathioprine	6 months–4 years versus >4 years		Yes

Kim et al. [28]	1999	CD	6-Mercaptopurine		<1 year versus 1-2 years versus 2-3 years versus >3 years	No

Holtmann et al. [29]	2006	CD	Azathioprine/6-mercaptopurine		<3 years versus 3-4 years versus >4 years	No

Treton et al. [18]	2009	CD	Azathioprine		Median 62 months versus 80 months	No

**Table 5:** Risk factors of relapse after drug withdrawal (HR: hazard ratio; RR: relative risk; OR: odds ratio; CI: confidence intervals). Results originate from multivariable analysis, except from [20, 30], where the univariate analysis identified only one risk factor in each group.

Authors	Year	Medication	Disease	Predictive of (while on therapy)			Predictive of (after drug withdrawal)		
				Relapse	Risk (when available)	Sustained remission	Relapse	Risk (when available)	Sustained remission
Hawthorne et al. [12]	1992	Azathioprine	UC					HR (one year older): 0.95; 95% CI: 0.93–0.98	Older age
								HR: 0.97; 95% CI: 0.93–1.01; *p* = 0.1	Longer duration of remission at entry

Cassinotti et al. [27]	2009	Azathioprine	UC			Concomitant aminosalicylates	Duration of azathioprine therapy (3–6 months versus >48 months)	HR: 2.78; 95% CI: 1.27–6.11	
							No remission versus remission	HR: 2.35; 95% CI: 1.43–3.85	
							Disease extent: left sided versus extensive colitis	HR: 1.79; 95% CI: 1.06–3.02	
							Proctosigmoiditis versus extensive colitis	HR: 2.02; 95% CI: 1.10–3.72	

Bouhnik et al. [16]	1996	Thiopurine	CD	Female gender	RR: 2.3; 95% CI: 1.0–5.1		Male gender	RR: 5.2; 95% CI: 2.2–12.0	
				Age of ≤26 years	RR: 2.5; 95% CI: 1.3–4.5				
							Duration of remission (<4 years)	RR: 6.6; 95% CI: 2.7–16.2	

Lémann et al. [15]	2005	Azathioprine	CD				CRP ≥ 20 mg/L	RR: 16.9; 95% CI: 2.7–104.3	
							Hemoglobin ≤ 12 g/dL	RR: 8.7; 95% CI: 1.6–48.8	
							Longer time without steroids (≥50 months)	RR: 5.2; 95% CI: 1.5–18.1	

Treton et al. [18]	2009	Azathioprine	CD				CRP ≥ 20 mg/L	HR: 58.6; 95% CI: 7.5–457	
							Neutrophil count ≥ 4 × 10^9^/L	HR: 3.2; 95% CI: 1.6–6.3	
							Hemoglobin ≤ 12 g/dL	HR: 4.8; 95% CI: 1.7–13.7	

Kim et al. [28]	1999	6-Mercaptopurine	CD				Younger age	1 year increase in age = 2.8% decrease of HR (95% CI: 1.0–4.8%)	
							Higher mercaptopurine doses	1 mg increase in remission doses = 1.8% increase of HR 1.8% (95% CI: 0.7–2.8%)	

Farkas et al. [24]	2013	Infliximab	UC				Previous cycle of infliximab therapy		

Domènech et al. [20]	2005	Infliximab	CD				Partial response in luminal CD after three infusions		
							Perianal disease in luminal and perianal CD after 1 year treatment		

Steenholdt et al. [30]	2012	Infliximab	CD				Longer disease duration at first infusion (median: 7 vs. 5 yrs)	HR: 1.1; 95% CI: 1.0–1.1	

Louis et al. [22]	2012	Infliximab	CD				Male gender	HR: 3.7; 95% CI: 1.9–7.4	
							Corticosteroid use in the preceding 6–12 months	HR: 3.5; 95% CI: 1.1–10.7	
							Infliximab trough levels ≥ 2 mg/mL	HR: 2.5; 95% CI: 1.1–5.4	
							CDEIS > 0	HR: 2.3; 95% CI: 1.1–4.9	
							Leukocyte count > 6 × 10^9^/L	HR: 2.4; 95% CI: 1.2–4.7	
							Hemoglobin ≤ 145 g/L	HR: 6.0; 95% CI: 2.2–16.5	
							CRP ≥ 5 mg/L	HR: 3.2; 95% CI: 1.6–6.4	
							Fecal calprotectin ≥ 300 *μ*g/g	HR: 2.5; 95% CI: 1.1–5.8	
							Absence of surgical resection	HR: 4.0; 95% CI: 1.4–11.4	

Molnár et al. [23]	2013	Infliximab or adalimumab	CD				Previous biological therapy	OR: 4.23; 95% CI: 1.39–12.84	
							Dose-intensification during the 1-year anti-TNF therapy	OR: 12.96; 95% CI: 1.39–120.5	
							Corticosteroid use at the beginning of anti-TNF therapy (in patients not receiving steroids)		
							Smoking status (in patients not receiving steroids)		
							Male gender (if patients with dose-intensification are excluded)	OR: 2.92; 95% CI: 1.06–8.2	
